# Anatomic variant of the internal carotid artery in the pharynx

**DOI:** 10.5935/1808-8694.20130142

**Published:** 2015-10-08

**Authors:** Amaury de Machado Gomes, Otavio Marambaia dos Santos, Pablo Pinillos Marambaia, Carlos Augusto de Carvalho Carrera, Leonardo Marques Gomes

**Affiliations:** a MSc in Medicine and Human Health - EBMSP; Specialist in Otorhinolaryngology Sleep Medicine and Traffic Medicine (Medical Preceptor of the ENT Internship Program at INOOA/ABORL-CCF); b Professor of Otorhinolaryngology, Bahiana School of Medicine - EBMSP; Doctoral Student, Porto School of Medicine - Portugal (Head of the ENT Service, Coordinator of the ENT Internship, INOOA/ABORL-CCF); c MSc in Medicine and Human Health - EBMSP; Specialist in Otorhinolaryngology (Medical Preceptor of the ENT Internship Program at INOOA/ABORL-CCF); d MD, EBMSP (Medical Intern, ENT Internship, INOOA/ABORL-CCF); e MD, EBMSP (ENT Medical Intern, NOSP/ABORL-CCF); ENT Associates - Otorhinolaryngology Institute - INOOA

**Keywords:** carotid artery, internal, pharynx, sleep apnea, obstructive, snoring

## INTRODUCTION

The internal carotid artery (ICA) moves cranially towards the skull base and does not branch out1., 2., 3.. The ICA arises from the bifurcation of the common carotid artery^4^.

Abnormalities in the course of the ICA may be present in 10%-40% of the general population1., 2., 3.. Frequent anomalies include tortuosity, kinking, and coiling1., 3., 5.. These may be congenital or secondary to senile alterations of the vascular tunic or atherosclerosis1., 2., 3., 4.. The alterations are usually asymptomatic2., 3., 6., but symptoms such as sleep disorders may occur^6^.

Aberrant internal carotid artery must be considered in the assessment of candidates for surgery in the pharyngeal space1., 2., 3., 4. such as adenotonsillectomy, once injuries to the ICA usually lead to severe hemorrhage1., 2., 3., 4..

This paper reports the case of an elderly patient with a pulsating mass in the rhinopharynx.

## CASE REPORT

MS, male, 79, came to our service complaining of nocturnal snoring, choking episodes and mouth breathing, all growing in intensity for the past six months. He had no other complaints and claimed not to have comorbidities.

Examination showed he had a BP of 130 × 80 mmHg, a body mass index of 26 Kg/m^2^, and a neck circumference of 42 cm. His ENT examination did not reveal significant alterations.

**Figure 1:**
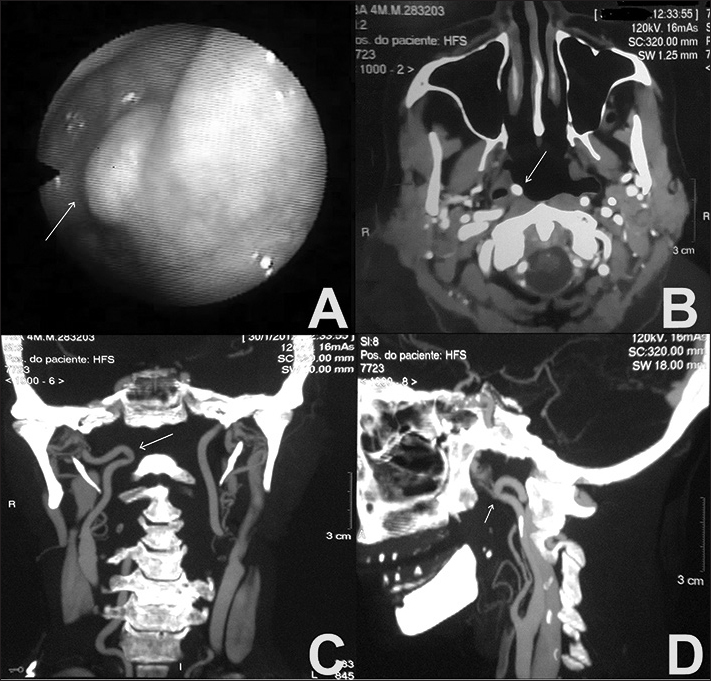
A: Nasal endoscopy image showing a pulsating mass in the lateral recess of the nasopharynx. B, C, D: Contrast-enhanced CT scan of the neck; axial, coronal, and sagittal views, respectively, showing a kinked right internal carotid artery.

The patient underwent overnight polysomnography (PSG) and was diagnosed with moderate obstructive sleep apnea syndrome (OSAS) - (apnea hypopnea index [AHI]: 16.2 events per hour). Nasal endoscopy with a flexible endoscope revealed a smooth mass in the rhinopharynx located in the right lateral recess pulsating synchronously with the patient's radial pulse (Figure 1A).

Contrast-enhanced CT scans of the neck showed a tortuosity in the right ICA and a kinked segment five centimeters away from the carotid artery bifurcation pressing against the right posterolateral wall of the rhinopharynx, with no evidences of significant airway amplitude reduction (Figures 1 B, C, D). Respiratory events were repaired (AHI: 2.1 events per hour) with the use of a continuous positive airway pressure device (nasal CPAP) of 6 cm H_2_O. The vascular surgeon preferred to wait and see how the patient would evolve.

## DISCUSSION

Examination findings indicated the kinking seen in the patient's ICA was an acquired condition. Despite the telltale signs of OSAS, these findings appeared to be unrelated to the bulging of the rhinopharynx, and were corrected with the use of CPAP. CT scans also suggested the anomaly did not cause the patient to have OSAS, as no significant narrowing of the airways was observed.

As also described by other authors, ICA alterations may be insidious2., 3., 6. and have close ties with the pharyngeal space. ENT physicians and head and neck surgeons must pay special attention to this fact, as injuries to the ICA may have catastrophic consequences. Patients prescribed surgery in sites where the ICA is present, including children scheduled to undergo adenotonsillectomy, should be systematically examined through nasal endoscopy with a flexible endoscope. Endoscopy is also a highly valuable diagnostic test to check for masses and rhinopharynx bulging in snorers, patients suffering from apnea episodes, and individuals prescribed CPAP. When such lesions are found, imaging tests such as computerized tomography, magnetic resonance imaging, and angiography are recommended for a more accurate assessment3., 4., 5., 6..

## CLOSING REMARKS

Aberrant ICA is usually asymptomatic. Subjects afflicted by upper airway obstruction, individuals diagnosed with OSAS, and patients scheduled for surgery in the parapharyngeal space must be systematically examined through nasal endoscopy with a flexible endoscope. Imaging plays a key role in the characterization of the anomalies detected in this site. No causality was found between ICA kinking and OSAS in the patient described in this case report.
